# Supported: Supporting, enabling, and sustaining homecare workers to deliver end-of-life care: A qualitative study protocol

**DOI:** 10.1371/journal.pone.0291525

**Published:** 2023-12-13

**Authors:** Zana Bayley, Joan Bothma, Alison Bravington, Cat Forward, Jamilla Hussain, Jill Manthorpe, Mark Pearson, Helen Roberts, Paul Taylor, Liz Walker, Caroline White, Jane Wray, Miriam J. Johnson

**Affiliations:** 1 Faculty of Health Sciences, Hull York Medical School, University of Hull, Hull, United Kingdom; 2 Cera Care, Laindon, Essex, United Kingdom; 3 King’s College London, London, United Kingdom; 4 Bradford Teaching Hospitals Foundation Trust, Bradford, United Kingdom; 5 School of Health and Related Research, University of Sheffield, Sheffield, United Kingdom; 6 St Luke’s Hospice, Sheffield, United Kingdom; UNITED KINGDOM

## Abstract

**Background:**

Homecare workers provide essential care at home for people at end-of-life but are often poorly trained and supported.

**Aim:**

To explore the experiences and needs of homecare workers and the views of homecare clients and carers, and other community-based health and social care staff about the homecare worker role, including identification of good practice.

**Methods:**

In this qualitative exploratory study, we will conduct 150 semi-structured interviews with homecare workers within three geographic English localities chosen for maximum socio-demographic variation. Eligible participants will be consenting adults providing care services (workers [n = 45], managers [n = 15] community practitioners [n = 30]), receiving care (clients thought to be in the last 6 months of life [n = 30], family carers [n = 15], or commissioners of homecare services supporting end-of-life care [n = 15]. Interviews may adopt a Pictor-guided or standard semi-structured approach according to their preference. Managers and commissioners can contribute to an online focus group if preferred. A range of recruitment strategies will be used, including through homecare agencies, local authorities, local NHS services, charities, voluntary sector groups and social media. Interviews and focus groups will be recorded, transcribed, anonymised, and analysed adopting a case-based approach for each geographic area within-case and then comparison across cases using reflexive thematic analysis. The design and analysis will be informed by Bronfenbrenner’s Adapted Ecological Systems theory. This study is registered on the Research Registry (No.8613).

**Contribution:**

We will provide evidence on ways to improve the experiences and address the needs of homecare workers in relation to caring for people nearing end-of-life. It will offer insight into good practice around supporting homecare workers including responding to their training and development needs. Findings will inform subsequent stages of an evaluation-phase study of a training resource for homecare workers.

## Introduction

Social homecare support enables people to stay at home and live well during illness, meets other care and support needs, helps prevent avoidable residential care or hospital admissions, and can enable a good death at home [1–3]. Despite the importance of the role, we know little about what skills and support are necessary to enable homecare workers (HCWs) to deliver high quality end-of-life (EoL) care at home.

HCWs support many people with care needs and any family or friends providing informal care (subsequently referred to as carers), often working alongside, but separate to, other health and social care providers. However, they often have little training, particularly in EoL care, and are poorly supported [2, 4–7]. In England, HCWs are primarily employed within commercial companies and have low status, low pay, limited career progression, isolated working conditions and employment insecurity. There are also specific challenges for HCWs who support people at EoL including negotiating emotional attachments, bereavement, role ambiguity, and maintaining professional boundaries [2, 4, 5]. These findings are mirrored internationally [8–11].

A review of service delivery models for older people at EoL found little reference to social care [12], indeed, little research has been undertaken with HCWs. There is a substantial evidence gap concerning the best way to support HCWs to provide sustainable, quality, integrated care for those requiring EoL care at home. Therefore, we aim to explore the experiences and needs of HCWs and the views of homecare clients and carers, and other health and social care staff, about the HCW role; identifying good practice and training gaps and needs, as well as ways to include and support HCWs within the wider care team supporting the client/carer.

This protocol forms one work package of a larger study exploring the experiences and needs of HCWs, with an overall aim to improve the quality and sustainability of person-centred EoL care by HCWs through training, skill development and empowerment, and to inform employment practices, commissioning, and policy.

## Methods

This qualitative study takes a social constructionist perspective. This views knowledge and meaning as constructed within social interactions and is sensitive to the geographical and temporal contexts within which it is produced. Bronfenbrenner’s Adapted Ecological Systems Theory [13] is the strategic theoretical framework informing this study design and analysis. This recognises five inter-related systems which capture the multiple, complex individual and service-level interactions which evolve over time within the dynamics of families, communities and society, informing a whole systems approach to EoL care and palliative care. The five systems relate to the person, their immediate environment, multidisciplinary care, local geographies and the overarching policy, funding and social/cultural context.

### Study design

In this qualitative exploratory study, we will conduct 150 in-depth, face-to-face or remote semi-structured interviews across community health and social care services in three English localities. We will use a pragmatic paradigm to define our approach to the local areas: the DESCARTE (Design of Case Study Research in Health Care) case study model, with each geographical locality comprising a case or ‘entity,’ and interviews taking place concurrently within each [14].

Case studies facilitate data collection and analysis within and across settings to produce transferable findings, offering a flexible approach enabling holistic enquiry from multiple perspectives [14, 15]. Each of the three geographical localities, the cases, have been chosen to represent diversity in respect of key demographic factors such as ethnicity and cultural diversity, urban and rural locations, affluence and deprivation, and age profile of the populations. These factors have been shown to influence uptake of and access to health and social care; people from more deprived communities, minoritized ethnic communities and older adults experience greater disadvantage than the general population regarding both an increased need for but reduced access to healthcare [16]. This will also enable a diverse cohort of participants from different backgrounds. The purpose of the case study approach is to explore commonalities and divergence between the localities in how HCWs are incorporated into service provision for people at EoL and to enable sensitivity to how the contextual factors above affect the giving and receiving of this care, to enable wider applicability of our study findings [15, 17].

The study has been designed with, and is receiving ongoing support from, a service user and carer advisory group (SUC) (n = 6) and a HCW advisory group (n = 3). These groups will provide advice and guidance throughout the study on language and communication, recruitment, ethical issues, and dissemination. The HCW advisory group will also input into the co-design workshops, and both groups will help to sense check emerging findings.

### Participants

Eligible participants will be consenting staff providing care (HCWs [n = 45], their managers [n = 15], community practitioners [n = 30]), and commissioners of homecare provision supporting EoL care in clients’ homes [n = 15]), people receiving care who are thought to be in the last 6 months of life [n = 30], and their carers [n = 15]. Clients will be identified by the relevant professional responsible for their care according to their opinion and use of the “surprise question” “I would not be surprised if this person died within the next 12 months” [18]. Participant groups, sample sizes and eligibility criteria are shown in Table 1.

**Table 1:** Participant groups, target recruitment, and eligibility criteria.

Participant Group	Recruitment No.	Inclusion Criteria	Exclusion Criteria
Homecare Workers (HCW)	15 per site–total 45	Providing end-of-life (EoL) care as part of their role or have provided care for one or more clients at EoL in the last 12 months	HCW has not had recent experience of delivering EoL care (in last 12 months)
Clients	10 per site–total 30	Individuals in receipt of homecare and who have been identified by their care provider as in the last six months of expected life Have capacity to consent to participation verbally or in writing Able to participate in interview for approx. 30–60 minutes Have sufficient English communication skills to participate, or be able to participate with the assistance of a communication assistant or interpreter	Anticipated to die within next 7 days as reported by homecare provider or health practitioner
Carers	5 per site–total 15	Carers (family/friend) of a client at EoL who is currently or previously receiving homecare Former carers at least 3 months after bereavement and no more than 12 months after bereavement Have sufficient English communication skills to participate, or be able to participate with the assistance of a communication assistant or interpreter	Carers of a person who is anticipated to die within the next 7 days, as reported by home care provider or health practitioner Former carers less than 3 months after bereavement or more than 12 months after bereavement
Community Practitioners	10 per site–total 30	Health and Social Care Practitioners who support people at EoL (or have done in last 12 months) e.g. district/community nurses, GPs (family physicians), palliative care practitioners, social workers	Practitioners who do not have current or recent (within last 12 months) experience of supporting people at EoL
Homecare Managers and Local Authority Commissioning Managers	10 per site–total 30	Working to provide and/or commission the delivery of homecare, including EoL care, or had role within last 12 months	Managers and commissioners who do not have a current or recent role (within last 12 months) providing and /or commissioning homecare services at EoL

A range of recruitment strategies will be used, including through homecare agencies, local authorities, local NHS providers, other health services, charities, voluntary sector groups and social media. Agencies will be identified using publicly available information and existing contacts and networks. Purposive sampling will primarily be used to achieve a diverse sample in respect of ethnicity, age, gender, socio-economic status, urban/rural location, funder of care (local authority or other), and employment status. However, for homecare managers and commissioners, convenience sampling will be adopted to include local authority providers (where possible), commercial, and not-for-profit agencies. HCWs will be recruited through homecare agency managers across the three sites, in addition to using a snowballing technique following completed interviews with other HCWs, and through the research units’ social media. Clients and carers may be identified through homecare agencies, as well as carer support groups and carer centres locally. Community practitioners will be recruited through agencies, social media and direct contact with healthcare practices such as GP clinics and community health teams. Commissioners will be identified through information in the public domain and local authority contacts within the research team.

Participants will have as much time as they need before providing consent, and an opportunity to ask questions. Witten or verbal consent will be taken prior to data collection. Capacity to participate will be presumed, as required by the Mental Capacity Act 2005 [19]. If necessary, this will be assessed by the researcher before and during the interview, and action taken if it is felt that capacity has diminished to the extent that consent to participate is no longer valid, by suspending or ending the process.

### Data collection

Data will be collected primarily through interviews which will use the Pictor technique–a simple guided diagramming technique which encourages participants to consider a real, lived event [20]. The technique is derived from personal construct psychology [17] and has been used successfully with patients, carers, and clinicians in research exploring service provision around advanced disease and EoL. Pictor aids recall and explanation, and reduces the intensity of the interview experience because the focus is on the diagram rather than the interview participant [21–23]. It has also been used to overcome noted barriers in research participation by those with advanced disease [24]. Participants will be invited to make a simple diagrammatic representation of their support network or an episode of care using arrow stickers to represent the interaction of the roles of those involved–the proximity and direction of the arrows can be used to describe aspects of that social interaction. On completion, participants will be asked to ‘tell the story’ behind their diagram. In HCW interviews, they will be encouraged to focus on barriers and facilitators to delivering good care and support. Clients and carers will be asked what factors they perceive would enable others to help them better and what fosters good care from HCWs and other practitioners. A semi-structured verbal interview without diagramming will be offered in the case of participants not willing, or able, to use Pictor.

Data collection will be conducted face to face where possible. However, if participants prefer, or in event of any social restrictions, interviews may be conducted online or *via* telephone. If conducted remotely, Pictor diagrams may be completed prior to the online interview where possible, if the participant chooses to do so. Prior to and during data collection, any communication needs such as interpreters or alternative forms of communication will be facilitated, and capacity to participate will be monitored throughout as necessary. If a carer and client wish to be interviewed together, this will be accommodated; Pictor charts will be undertaken separately with each participant.

Agency managers and commissioners can opt to participate in a focus group rather than interview, which will be accommodated where possible without the Pictor technique. Data from any focus groups will be analysed cognisant of the differences with individual interviews and how that may impact on data collected and identifiable codes.

Separate interview topic schedules will be developed for each participant group for both individual and group interviews (see S1 File for an example schedule). These will explore participant perspectives on care delivered, HCWs’ support needs, HCWs’ training needs and the provision available, personal and professional networks for HCWs, workforce retention, coordination and continuity of care for clients, and any difficulties and challenges in assuring this, and will be further developed by issues arising in ongoing analysis. All data will be stored securely within university systems.

Any participant appearing distressed during an interview will be offered the opportunity to pause or withdraw from the study and signposted to local and national sources of support as necessary. Researchers will have access to a developed support network within the expertise of the research team.

Interviews and focus groups will be audio-recorded, transcribed, and anonymised for confidentiality by a university-authorised transcription company with a confidentiality agreement in place. Pictor charts will be photographed; if participants use names on Pictor charts, these will be anonymised before photographing.

### Data management

This study will be conducted in compliance with the protocol, the Declaration of Helsinki [25], the UK Policy Framework for Health and Social Care Research [26], the Data Protection Act 2018 [27] and General Data Protection Regulations [28] and other regulatory requirements as appropriate. All data will be stored securely within university systems.

### Data analysis

Analysis will adopt a case-study based approach [14], with initial analysis focusing on each geographical locality (case) in turn and subsequent analysis focusing on comparisons across sites, and take account of the five inter-related systems from Bronfenbrenner’s Adapted Ecological Systems Theory [13]. Within each case, interview and focus group data will be analysed separately initially, using reflexive thematic analysis following Braun and Clark’s stages of data familiarisation, code formation and development of themes [29] as a collaborative process within the research team. Pictor charts from the individual interviews, where completed, will be analysed by exploring patterns such as the frequency with which specific roles appear and the formation of charts in network or timeline arrangements. This will enable us to re-interrogate the interview data in relation to diverse roles of the participants [23]. The interpretation of the interaction between the graphical, i.e. Pictor charts, and the textual, i.e. interviews data, in all rounds of analysis will be led by an expert in visual methods from the research team (AB).

At this stage, study findings will be discussed with the service user and homecare worker representatives to explore the resonance of the themes, and feedback integrated into, and influencing, the study outcomes [30]. Matrix analysis will then be used to provide a cohesive approach to cross-case comparisons [31], with an *a priori* focus on the five overarching systems of Bronfenbrenner’s Adapted Ecological Systems Theory [13].

Findings will also be presented to other stakeholders identified in Pictor charts or partnering with the research team (for example Skills for Care–the national strategic workforce development and planning body for adult social care in England) to inform and structure our dissemination plans.

### Ethical considerations

This protocol underpins a work package from a larger study entitled “SUPPORTED: Supporting, enabling and sustaining homecare workers to deliver end-of-life care: A multiple-methods community-based case study.” This protocol was approved by the Health Research Authority (HRA) West Midlands Research Ethics Committee on 31^st^ March 2023 ref 23/WM/0030.

Information sheets for each group will be supplied for consideration and questions or discussion prior to consent being requested. Care was taken over the participant-facing wording relating to the eligibility criterion relating to prognosis as clients receiving homecare at the EoL may not be aware of their short prognosis. Approved wording was agreed in conjunction with our advisory groups and the HRA Research Ethics Committee. Signposting for psychological support will be made available to participants if needed.

## Discussion

Care at home is a crucial component of person-centred care, enabling choice and preferences to be adopted within a culture of patient-or person-directed care, particularly at EoL, where it is evidenced that most people would prefer to receive care within their home environment [31]. Within England and many other high-income nations, the delivery of this provision is supported by HCWs. Any improvements to this care need to be informed by this important workforce and those connected to that provision.

### Contribution

This study will fill a gap in knowledge relating to HCWs’ experiences of supporting people at EoL. This is vital for societies where home may be the preferred place of care for many people when faced with an advanced non-curative condition and for ‘ageing in place’ more generally.

Findings from this study will help us to understand the lived experience of HCWs working with people towards the EoL, the challenges they face, and their needs in relation to providing that care. This understanding will be comprehensive, including perspectives not only of HCWs themselves, but also of people with advanced illness receiving homecare, their carers, homecare managers, commissioners, and community practitioners. Findings will provide in-depth understanding of HCW’s training needs; psychological and emotional support and reward; practice challenges; and inform the content of training materials.

We will use a novel approach with Pictor diagramming; a technique shown to be appropriate in the context of EoL research. Pictor has been used to enable in-depth conversations and generate a more holistic discussion [23, 24, 32], enabling a wider scope for generating understanding across multiple perspectives.

### Dissemination

This study will inform the larger project and use the findings alongside evidence from local and national policy and guidance to investigate options for resources to better equip HCWs to feel competent and confident in delivering this service. Results will be presented and published for the wider health and care and research audience to enable this data to influence not only conversation and debate around supporting HCWs more broadly, but to encourage changes and improvements culturally and practically worldwide.

### Study limitations

The study has limitations in that is it primarily focusing on homecare provision within England. The study has been designed around three different localities to ensure a wide range of communities and backgrounds, however other countries may adopt different care models which may not have the same focus on the commissioning of private sector services. Also, a significant part of care for people approaching EoL may be provided through a national or local health service, and other health agencies which are not considered in this study. How those services may interact with HCWs working alongside other practitioners is an aspect that will be discussed in the interviews where appropriate. We also acknowledge the significant number of carers, such as family and friends, who provide care [33], and are often not considered when training for HCWs is developed; and incorporate this often overlooked perspective [34] However, there are considerably greater numbers of studies focusing on family carers and their needs [35–37]. As such this study focuses on the provision by HCWs which does not receive as much attention.

## Supporting information

S1 File Interview guide example. (DOCX)

S2 File Consent form example. (DOCX)

## References

[1] Care Quality Commission (CQC). Not Just a Number. Home Care Inspection Programme. 2021. CQC. https://www.cqc.org.uk/sites/default/files/documents/9331-cqc-home_care_report-web_0.pdf

[2] Abrams R, Vandrevala T, Samsi K, Manthorpe J. The need for flexibility when negotiating professional boundaries in the context of home care, dementia and end of life. Ageing and Society 2018; 39:1976–95. doi: 10.1017/s0144686x18000375

[3] Abe K, Miyawaki A, Kobayashi Y, Noguchi H, Takahashi H, Tamiya N. Receiving the home care service offered by certified care workers prior to a patients’ death and the probability of a home death: observational research using an instrumental variable method from Japan. BMJ Open 2019;9:e026238. doi: 10.1136/bmjopen-2018-026238 31462462 PMC6719756

[4] Schneider J, Pollock K, Wilkinson S, Perry-Young L, Travers C, Turner N. The subjective world of home care workers in dementia: an “order of worth” analysis. Home Health Care Services Quarterly 2019;38:96–109. doi: 10.1080/01621424.2019.1578715 30794075

[5] Leverton M, Burton A, Beresford-Dent J, Rapaport P, Manthorpe J, Mansour H, et al. ‘You can’t just put somebody in a situation with no armour.’ An ethnographic exploration of the training and support needs of homecare workers caring for people living with dementia. Dementia 2021;20:2982–3005. doi: 10.1177/14713012211023676 34111969 PMC8678657

[6] Fenton W, Polzin G, Price R, et al. The state of the adult social care sector and workforce in England. Leeds. 2021. Skills for Care, https://www.skillsforcare.org.uk/adult-social-care-workforce-data/Workforceintelligence/publications/national-information/The-state-of-the-adult-social-care-sector-and-workforce-in-England.aspx

[7] Bottery S. (Ed). Home care in England. Views from commissioners and providers. 2018 London. The King’s Fund. https://www.kingsfund.org.uk/sites/default/files/2018-12/Home-care-in-England-report.pdf

[8] Tsui EK, Wang W-Q, Franzosa E, Gonzalez T, Reckrey JM, Sterling MR, et al. Training to Reduce Home Care Aides’ Work Stress Associated with Patient Death: A Scoping Review. Journal of Palliative Medicine 2020;23:1243–9. doi: 10.1089/jpm.2019.0441 31855094

[9] Percival J, Lasseter G, Purdy S, Wye L. “Earthly Angels”? A qualitative study of the domiciliary care worker role in meeting the needs of families caring for those dying at home. Palliative and Supportive Care 2013;12:445–53. doi: 10.1017/S147895151300076X 24138887

[10] Manson J, Gardiner C, Taylor P, Ghasemi L, Westerdale-Shaw E, Sutton L, et al. ‘Palliative care education in nursing homes: a qualitative evaluation of telementoring. BMJ Supportive & Palliative Care 2021 doi: 10.1136/bmjspcare-2020-002727 33627368

[11] Fukui S, Fujita J, Ikezaki S, Nakatani E, Tsujimura M. Effect of a multidisciplinary end-of-life educational intervention on health and social care professionals: A cluster randomized controlled trial. PLOS ONE 2019;14:e0219589. doi: 10.1371/journal.pone.0219589 31425538 PMC6699737

[12] Evans CJ, Ison L, Ellis-Smith C, Nicholson C, Costa A, Oluyase AO, et al. Service Delivery Models to Maximize Quality of Life for Older People at the End of Life: A Rapid Review. The Milbank Quarterly 2019;97:113–75. doi: 10.1111/1468-0009.12373 30883956 PMC6422603

[13] Pask S, Pinto C, Bristowe K, van Vliet L, Nicholson C, Evans CJ, et al. A framework for complexity in palliative care: A qualitative study with patients, family carers and professionals. Palliative Medicine 2018; 32(6), 1078–1090. doi: 10.1177/0269216318757622 29457743

[14] Carolan CM, Forbat L, Smith A. Developing the DESCARTE Model. Qualitative Health Research 2015;26:626–39. doi: 10.1177/1049732315602488 26336896

[15] Paparini S, Green J, Papoutsi C, Murdoch J, Petticrew M, Greenhalgh T, et al. Case study research for better evaluations of complex interventions: rationale and challenges. BMC Medicine 2020;18. doi: 10.1186/s12916-020-01777-6 33167974 PMC7652677

[16] Marmot M, Allen J, Boyce T, Goldblatt P, Morrison J. Health Equity in England: The Marmot Review 10 Years On. Institute of Health Equity; 2020 (health.org.uk/publications/reports/the-marmot-review-10-years-on)

[17] Yin RK. Case Study Research: Design and Methods. 2008. doi: 10.1604/9781412960991

[18] Murray S and Boyd K. Using the ’surprise question’ can identify people with advanced heart failure and COPD who would benefit from a palliative care approach. Palliative medicine 2011; 25:382 doi: 10.1177/0269216311401949 21610113

[19] Mental Capacity Act 2005. UK. Available at https://www.legislation.gov.uk/ukpga/2005/9/contents

[20] Bravington A, King N. Putting graphic elicitation into practice: tools and typologies for the use of participant-led diagrams in qualitative research interviews. Qualitative Research 2018;19:506–23. doi: 10.1177/1468794118781718

[21] Burr V, King N and Butt T. Personal construct psychology methods for qualitative research. Int J Soc Res Methodology 2014; 17:4 341–355. doi: 10.1080/13645579.2012.730702

[22] Bulk LY, Kimel G, King N, Nimmon L. Understanding Experiences in Hospice: Exploring Temporal, Occupational, and Relational Dimensions Using Pictor Technique. Qualitative Health Research 2020; 30:1965–77. doi: 10.1177/1049732320926134 32564687

[23] King N, Bravington A, Brooks J, Hardy B, Melvin J, Wilde D. The Pictor Technique. Qualitative Health Research 2013; 23:1138–52. doi: 10.1177/1049732313495326 23774630

[24] Hardy B, King N, Firth J. Applying the Pictor technique to research interviews with people affected by advanced disease. Nurse Researcher 2012;20:6–10. doi: 10.7748/nr2012.09.20.1.6.c9302 23061268

[25] Helsinki Declaration 1996. South Africa. Available at https://www.wma.net/what-we-do/medical-ethics/declaration-of-helsinki/doh-oct1996/

[26] Department of Health. UK Policy Framework for Health and Social Care Research. 2023. Available at https://www.hra.nhs.uk/planning-and-improving-research/policies-standards-legislation/uk-policy-framework-health-social-care-research/uk-policy-framework-health-and-social-care-research/

[27] Data Protection Act 2018. UK. Available at https://www.legislation.gov.uk/ukpga/2018/12/contents/enacted

[28] General Data Protection Regulation 2018. EU. Available at https://gdpr-info.eu/

[29] Braun V, Clarke V. Reflecting on reflexive thematic analysis. Qualitative Research in Sport, Exercise and Health 2019;11:589–97. doi: 10.1080/2159676x.2019.1628806

[30] Boaz A, Hanney S, Borst R, O’Shea A. and Kok M. How to engage stakeholders in research: design principles to support improvement. Health Res Policy Sys 2018; 16:60 doi: 10.1186/s12961-018-0337-6 29996848 PMC6042393

[31] Miles MB, Huberman M. Qualitative Data Analysis: An Expanded Sourcebook. 1994. doi: 10.1604/9780803946538

[32] Johnston B, Patterson A, Bird L, Wilson E, Almack K, Mathews G, et al. Impact of the Macmillan specialist Care at Home service: a mixed methods evaluation across six sites. BMC Palliative Care 2018;17. doi: 10.1186/s12904-018-0281-9 29475452 PMC6389143

[33] Swan S, Meade R, Cavers D, Kimbell B, Lloyd A and Carduff E. Factors influencing adult carer support planning for unpaid caregiving at the end of life in Scotland: Qualitative insights from triangulated interviews and focus groups. Health and Social Care in the Community 2022 30:4 1422–1432. doi: 10.1111/hsc.13472 34427355 PMC9290463

[34] Felstead C, Perkins L, Stott J, Hui EK, Spector A. A systematic literature review of group-based training interventions for informal carers: impact on the behavioural and psychological symptoms of dementia (BPSD). Aging & Mental Health 2022:1–10. doi: 10.1080/13607863.2022.2141193 36369837

[35] Grande G, Shield T, Bayliss K, Rowland C, Flynn J, Bee P, et al. Understanding the potential factors affecting carers’ mental health during end-of-life home care: a meta synthesis of the research literature. Health and Social Care Delivery Research 2022:1–62. doi: 10.3310/ekvl3541 36976892

[36] Raemdonck E, Lambotte D, De Witte N, Gorus E. Giving voice to informal caregivers of community‐dwelling older adults: A systematic review of empowerment interventions. Health & Social Care in the Community 2022;30. doi: 10.1111/hsc.13928 35899425

[37] Matthys O, Dierickx S, Deliens L, Lapeire L, Hudson P, Van Audenhove C, et al. How are family caregivers of people with a serious illness supported by healthcare professionals in their caregiving tasks? A cross-sectional survey of bereaved family caregivers. Palliative Medicine 2022;36:529–39. doi: 10.1177/02692163211070228 35090372

