# Correction: Supported: Supporting, enabling, and sustaining homecare workers to deliver end-of-life care: A qualitative study protocol

**DOI:** 10.1371/journal.pone.0298925

**Published:** 2024-02-12

**Authors:** Zana Bayley, Joan Bothma, Alison Bravington, Cat Forward, Jamilla Hussain, Jill Manthorpe, Mark Pearson, Helen Roberts, Paul Taylor, Liz Walker, Caroline White, Jane Wray, Miriam J. Johnson

There are errors in the Funding section. The correct Funding statement is: This study/project is funded by the NIHR HSDR Programme (project reference NIHR135128). The views expressed are those of the author(s) and not necessarily those of the NIHR or the Department of Health and Social Care. The funders did not and will not have a role in study design, data collection and analysis, decision to publish, or preparation of the manuscript.
