# Supplementary material for: Supported: Supporting, enabling, and sustaining homecare workers to deliver end-of-life care: A qualitative study protocol
Source: PLoS One. 2023 Dec 13;18(12):e0291525. doi: 10.1371/journal.pone.0291525 (PMC10718427; doi:10.1371/journal.pone.0291525)
Supplement: S1 File — (DOCX) [file pone.0291525.s001.docx]

**SUPPORTED: a study of** **the role of homecare workers in supporting people living with advanced illness at home**

**Participant Information Sheet – Homecare Workers**

**Joint Lead Study Investigators – Professor Liz Walker and Professor Miriam Johnson**

Homecare workers play important roles in supporting people living with advanced illness approaching the last months of life. However, we know very little about their experiences of this role and their training and support needs.

The SUPPORTED study is researching how homecare workers support people living with advanced illness approaching the final months of life. At the end of the research we will develop a training resource for homecare workers.

We are inviting you to take part in this research

If you are a homecare worker who supports clients at home at end of life, or if you have provided this support in the last 12 months, we would like to invite you to take part in this research.

Please take the time to read this leaflet and discuss it with other people if you wish. You can ask the researchers for more information and ask them questions if anything is not clear. Their contact details are at the end of this leaflet.

Do I have to take part?

Whether or not you take part is completely up to you. Whatever you decide will not affect your employment in any way, or disadvantage you. We will not inform your employer whether or not you choose to take part.

What will I need to do?

If you decide to take part you will be invited to be interviewed by one of our researchers about your experiences. The interview will take place in your workplace, at home or in another suitable place. It could also take place online using a ‘secure online meeting room’ such as Zoom or Teams. The interview will take no more than one hour (and may take less). It will be at a time which is convenient to you. The questions will include:

- How you support people living at home with advanced illness approaching the last months of life
- How you work alongside their unpaid carers (e.g. family or friends)
- Your experiences of working with other health and social care professionals
- How and if other people support you in your role
- Any difficulties or benefits you experience when supporting people at the end of life and their carers
- Any training you have had – and any training you would like.

During the interview we will use a simple method to help you talk about your experiences. This involves the researcher helping you to build a simple picture or diagram using sticky Post-It™ notes to represent the other people you work with and the people you support. This is called a Pictor diagram. You will not be asked to draw anything! Most people find this way of working easier than answering a list of questions. You do not need to prepare anything before the interview. You can take part without using the picture method if you would prefer, and you will be asked some questions about your work by the researcher. The interview will be recorded with your permission (sound only for face to face interviews, or sound and video for online interviews).

If you would like to find out a bit more about what is involved in making a Pictor diagram, we made a short video which might help you decide about whether you want to make a Pictor diagram during the interview. You do not have to watch the video (the researcher will give you all the information you need), and you do not need to prepare anything ahead of the interview. You can watch the video at [link currently unavailable].

After the interview what you said will be typed up, and we will take out your name and the names of any other people and organisations (including your employer) that you mention. The person who types up the interview may be outside of the research team - in this case they will sign a confidentiality agreement to keep your information private.

During the interview you do not have to answer any questions you do not wish to answer or you cannot answer. You can stop the interview or take a break at any time. If you decide not to carry on with the interview, we will keep and analyse any information you have already shared. If talking about your experiences makes you feel upset in any way, you will be offered the option of taking a break or stopping the interview if you wish.

You will be given a £20 shopping voucher to thank you for sharing your valuable experience. Your employing agency will also be credited for one hour for your employment.

What will you do with the information from the research?

At the end of the research we will use the information shared by everyone who took part to develop a training resource for homecare workers. We will also write reports about what we found out, give talks, and write summaries to send to people and organisations who influence how homecare services are provided and supported. When we do this we will not use any information or details which would allow other people to identify you. We may write something you have said using your own words – but we will take out anything that would identify you, your employer or anyone else.

Who do I contact if I want to take part or if I have any questions?

You can contact the research team using the following contact details:

**The University of Hull researchers** (for people working in Hull, Bradford and the surrounding areas)

Email - [hcw-supported@hull.ac.uk](about:blank)

Phone - 07484 504863 (if in Hull) or 07872 420223 (if in Bradford)

**The King’s College London researchers** (for people working in south London)

Email - [catherine.forward@kcl.ac.uk](about:blank)

Phone - 07818 424 575

**For concerns or complaints** you can talk in the first instance to the research leads Professor Liz Walker ([E.Walker@hull.ac.uk](about:blank)) or Professor Miriam Johnson ([Miriam.Johnson@hyms.ac.uk](about:blank)).

If you wish to speak to someone from outside the research team please contact please contact the University of Hull Secretary and Chief Compliance Officer, Chris Ince at [university-secretary@hull.ac.uk](about:blank) Chris Ince is not a member of the research team.

Additional information about the research

**Ethical approval** for this study was given by the West Midlands - Coventry & Warwickshire Research Ethics Committee, 31^st^ March 2023, reference 23/WM/0030. Ethics Committees make sure that researchers treat people fairly and carry out research safely.

**Funding information.** This research is funded by the National Institute for Health and Care Research.

**Sponsor.** The Sponsor for the research is the University of Hull.

Please see the information overleaf to find out more about how we will use your information.

How will we use information about you?

- We will need to use information from you for this research project.
- This information will include your contact details, so that we can arrange an interview with you at your convenience. It will also include the words from your interview.
- People will use this information to do the research, and to check that the research is being done properly.
- People who do not need to know who you are will not be able to see your name or contact details. Your interview data will have a code number instead, and we will remove any words which might identify you.
- We will keep all information about you safe and secure.
- We will write our reports in a way that no-one can work out that you took part in the study. If we use quotes from your interview we will take out any information that would allow anyone else to identify you or other people.
- The only time that we will tell anyone else that you took part in the research is if you tell us anything that suggests you or someone else is being hurt, harmed or abused, is at risk, or you share information about poor practice. If this happens then we will have to share this information to make sure that any problems are addressed. This might include informing your employer or the Local Authority Safeguarding Team.
- All the information we collect for this study will be made available to other authorised researchers at the end of the study for additional research, including information you have already provided if you stop taking part in the study. However, information will only be shared for worthwhile research projects with appropriate ethical approvals, only in such a way that no individual person can be identified, and only when we are sure the other researchers will manage your data correctly and securely. Sharing of research data is an important way to make the most of the time and effort required to carry out research.

GDPR statement (Data Protection)

The data controller for this project will be the University of Hull. The University will process your personal data for the purpose of the research outlined above. The legal basis for processing your personal data for research purposes under GDPR (General Data Protection Regulation) is a ‘task in the public interest’

If you are not happy with the Sponsor’s response or believe the Sponsor is processing your data in a way that is not right or lawful, you can complain to the Information Commissioner’s Office (ICO) ([www.ico.org.uk](about:blank) or 0303 123 1113).

What are your choices about how your information is used?

- Taking part is voluntary. You can stop being part of the study at any time, without giving a reason. However, we will keep information about you that we already have.
- We need to manage your records in specific ways for the research to be reliable. This means that you won’t be able to change information you have already given to the study.

Where can you find out more about how your information is used?

You can find out more about how we use your information:

- At [www.hra.nhs.uk/information-about-patients/](about:blank)
- By asking one of the research team
- By contacting the research leads – Professor Liz Walker ([e.walker@hull.ac.uk](about:blank) or 01482 463336) or Professor Miriam Johnson ([Miriam.johnson@hyms.ac.uk](about:blank) or 01482 463442)
- By sending an email to the University of Hull’s Data Protection Officer at [dataprotection@hull.ac.uk](about:blank)

How can you find out about the results of the study?

We are planning to complete the study in 2025. The researcher can arrange to send you a report of the results after this date. A copy of the summary will be made available on the SPARC (Social and Psychological Research in Long Term Conditions) website at the University of Hull: [https://www.hull.ac.uk/work-with-us/research/groups/social-and-psychological-research-in-long-term-conditions-sparc](about:blank)
