# Supplementary material for: Supported: Supporting, enabling, and sustaining homecare workers to deliver end-of-life care: A qualitative study protocol
Source: PLoS One. 2023 Dec 13;18(12):e0291525. doi: 10.1371/journal.pone.0291525 (PMC10718427; doi:10.1371/journal.pone.0291525)
Supplement: S2 File — (DOCX) [file pone.0291525.s002.docx]

**SUPPORTED: a study of the role of homecare workers in supporting people living with advanced illness at home**

**Consent Form – Homecare Workers**

**Joint Lead Study Investigators – Professor Liz Walker and Professor Miriam Johnson**

Please initial the boxes below to indicate your consent:

|  | Initials |
| --- | --- |
| I have read the Information Sheet (version 2, dated 9^th^ March 2023). I have been able to ask questions and I am happy with the answers to these. |  |
| I understand that it is up to me whether I take part. I know that I can take a break and that I am free to withdraw from the research at any time without giving a reason. I understand that if I withdraw, the information I have already shared will be included in the research. |  |
| I agree that the researcher can record the interview (audio if in person, audio-visual for online interviews). I know that I can still take part in the research if I don’t agree to this. |  |
| I know that the researchers will not tell anyone that I took part in the research or put my name in any report. However, I understand that the person who types up the interview may be outside the research team (if I agree that the interview can be recorded). In this case I know that a confidentiality agreement will be in place. |  |
| I understand that if I disclose poor practice or safeguarding concerns, then the researchers will report these concerns, so that any problems can be addressed. In this case they will need to identify me. |  |
| I understand that the research data, which will be anonymised (not linked to me), will be retained by the researchers and may be shared with others and publicly disseminated to support other research in the future. |  |
| I agree that the researchers can include something I have said in my own words and in reports and publications. This will be without information that could identify anyone. I know I can still take part in the research if I don’t agree to this. |  |
| I agree that the researchers can include anonymised visual materials from my interview in publications and reports. This will be without information that could identify anyone. I know I can still take part in the research if I don’t agree to this. |  |
| I agree that the information collected about me may be used to support other research in the future, and may be shared anonymously with other researchers. |  |
| I agree to take part in this research |  |

Please sign below:

Name:

Date:

Signed:

Details of the person taking consent:

Name:

Date:

Signed:

Would you like to receive a copy of the final report of the research (we expect this will be available in 2025)? Yes/No

If yes, please give your preferred contact details below (email or post):

……………………………………………………………………………………………………………………………………………

……………………………………………………………………………………………………………………………………………

One copy to be retained by the participant, one copy to be retained by the research team
