# Supplementary material for: Supported: Supporting, enabling, and sustaining homecare workers to deliver end-of-life care: A qualitative study protocol
Source: PLoS One. 2023 Dec 13;18(12):e0291525. doi: 10.1371/journal.pone.0291525 (PMC10718427; doi:10.1371/journal.pone.0291525)
Supplement: Peer review history

# Editor report: Decision Letter 0

**DOI:** 10.1371/journal.pone.0291525.r001

**Authors:** Richard Ali

31 Aug 2023

SUPPORTED: SUPPORTING, ENABLING, AND SUSTAINING HOMECARE WORKERS TO DELIVER END-OF-LIFE CARE: A QUALITATIVE STUDY PROTOCOL

PONE-D-23-21979

Dear Dr. Bayley,

We’re pleased to inform you that your manuscript has been judged scientifically suitable for publication and will be formally accepted for publication once it meets all outstanding technical requirements.

Within one week, you’ll receive an e-mail detailing the required amendments. When these have been addressed, you’ll receive a formal acceptance letter and your manuscript will be scheduled for publication.

An invoice for payment will follow shortly after the formal acceptance. To ensure an efficient process, please log into Editorial Manager at http://www.editorialmanager.com/pone/, click the 'Update My Information' link at the top of the page, and double check that your user information is up-to-date. If you have any billing related questions, please contact our Author Billing department directly at authorbilling@plos.org.

If your institution or institutions have a press office, please notify them about your upcoming paper to help maximize its impact. If they’ll be preparing press materials, please inform our press team as soon as possible -- no later than 48 hours after receiving the formal acceptance. Your manuscript will remain under strict press embargo until 2 pm Eastern Time on the date of publication. For more information, please contact onepress@plos.org.

Kind regards,

Richard Ali, PhD

Staff Editor

PLOS ONE

Journal requirements:

When submitting your revision, we need you to address these additional requirements.

1. Please ensure that your manuscript meets PLOS ONE's style requirements, including those for file naming. The PLOS ONE style templates can be found at

https://journals.plos.org/plosone/s/file?id=wjVg/PLOSOne_formatting_sample_main_body.pdf and

https://journals.plos.org/plosone/s/file?id=ba62/PLOSOne_formatting_sample_title_authors_affiliations.pdf

# Editor report: Acceptance letter

**DOI:** 10.1371/journal.pone.0291525.r002

**Authors:** Richard Ali

25 Sep 2023

PONE-D-23-21979 

SUPPORTED: SUPPORTING, ENABLING, AND SUSTAINING HOMECARE WORKERS TO DELIVER END-OF-LIFE CARE: A QUALITATIVE STUDY PROTOCOL 

Dear Dr. Bayley:

I'm pleased to inform you that your manuscript has been deemed suitable for publication in PLOS ONE. Congratulations! Your manuscript is now with our production department. 

If your institution or institutions have a press office, please let them know about your upcoming paper now to help maximize its impact. If they'll be preparing press materials, please inform our press team within the next 48 hours. Your manuscript will remain under strict press embargo until 2 pm Eastern Time on the date of publication. For more information please contact onepress@plos.org.

If we can help with anything else, please email us at plosone@plos.org. 

Thank you for submitting your work to PLOS ONE and supporting open access. 

Kind regards, 

PLOS ONE Editorial Office Staff

on behalf of

Richard Ali 

Staff Editor

PLOS ONE
